# Establishment of Hematological and Plasma Biochemical Reference Values and Analysis of Risk Factors for Pet Sugar Gliders (*Petaurus breviceps*) in Taiwan

**DOI:** 10.3390/ani12243583

**Published:** 2022-12-18

**Authors:** Vivian C. Y. Lin, Ning-Ya Yang, Wen-Chi Lin, Jo-Wen Chen, Ching-Yi Yen, Yi-Lun Tsai

**Affiliations:** 1 Jong-Shing Mercy Animal Medical Center, Kaohsiung 81361, Taiwan; 2 Department of Veterinary Medicine, College of Veterinary Medicine, National Pingtung University of Science and Technology, Pingtung 91201, Taiwan; 3 Veterinary Medical Teaching Hospital, College of Veterinary Medicine, National Pingtung University of Science and Technology, Pingtung 91201, Taiwan

**Keywords:** sugar glider, *Petaurus breviceps*, hematology, plasma biochemistry, blood test, reference interval

## Abstract

**Simple Summary:**

Sugar gliders (*Petaurus breviceps*) are small, nocturnal, arboreal marsupials native to New Guinea and Australia, and are increasingly popular pets in Taiwan and the United States. Symptoms of common sugar glider diseases are often non-specific; therefore, blood tests, including hematology and plasma biochemistry, are important diagnostic tools for veterinarians. However, previous studies on this topic are rare. The goal of this study was to establish reference values of the aforementioned blood tests for pet sugar gliders. A total of 42 healthy pet sugar gliders were recruited, and hematological and plasma biochemical reference values were calculated. The influence of factors including age, gender, season and raising management were also analyzed. The information presented in this study can be beneficial for the future veterinary care of sugar gliders.

**Abstract:**

Hematological and plasma biochemical examination are crucial in the veterinary care of sugar gliders, which are increasingly popular in Taiwan and the United States. However, published research of the species’ reference interval and related influencing factors were rare. The objectives of this study were to establish the hematological and plasma biochemical reference values for captive sugar gliders in Taiwan and to evaluate the influence of factors including age, gender, neuter status, location, season, diet, caging arrangement, and other pets in the household. A total of 42 clinically healthy pet sugar gliders were recruited. Morphometrical measurements and physiological data were collected, and hematological and plasma biochemical examinations were performed. The reference value of each index was calculated using Reference Value Advisor (RVA) software, following the American Society for Veterinary Clinical Pathology (ASVCP) guidelines. Normality of data distribution was tested, and data transformation was conducted. The parametric method and robust method were used to determine reference limits. Univariate analysis was performed, and multiple regression models were built for each hematological and plasma biochemical parameter. Red blood cell, hematocrit, and hemoglobin levels were higher in males, compared to females, while they were lower in the neutered group, compared to the intact group. Relative neutrophil counts were higher in elder sugar gliders, while relative lymphocyte counts were lower. Aspartate transaminase levels were higher in elder sugar gliders, while albumin levels were lower. Blood urea nitrogen levels were highest in spring. The blood profile and related effects presented in this study can provide useful information for veterinary care in pet sugar gliders.

## 1. Introduction

Sugar gliders (*Petaurus breviceps*) are marsupials found in Australia and New Guinea [1,2]. Their lifespan is about 4 to 6 years in the wild, and 12 to 14 years in captivity [3,4,5]. Sugar gliders were first brought into the United States pet trade in the 1990s [6]. They are also a prominent part of the European and Asian exotic pet market [7]. Like many small mammal pets, sugar gliders tend to hide signs of disease, and are often seriously ill when brought to veterinary clinics [8,9]. Diagnostic tests, such as hematology and plasma biochemistry, are crucial for identifying underlying cause of the symptoms and to assess the severity of disease [8,10,11]. As Taiwan’s pet sugar glider population grows, the need for these examinations increases. However, studies on the hematological and biochemical reference intervals for sugar gliders are few, and none were conducted in Asia [4,6,10,12]. Further research is needed, as sugar gliders in Taiwan experience differences in climate, diet, and husbandry, which may influence their hematological and biochemical profile [13,14,15].

The aims of this study were to establish hematological and plasma biochemical reference intervals for pet sugar gliders in Taiwan and to evaluate the association between the parameters and risk factors, including age, gender, season, etc.

## 2. Materials and Methods

### 2.1. Study Population

**Figure 1:**
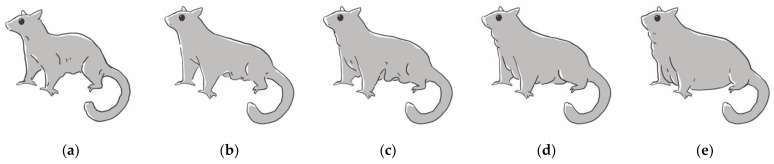
Reference figures for body condition scoring. (**a**) Score 1, no subcutaneous fat at neck/chest region, ribs easily palpable with no fat covering, thin patagium; (**b**) Score 2, minimal subcutaneous fat at neck/chest region, ribs easily palpable with little fat covering, thin patagium; (**c**) Score 3, slight subcutaneous fat at neck/chest region, ribs palpable with slim fat covering, thin patagium; (**d**) Score 4, thick subcutaneous fat at neck/chest region, ribs not easily palpable with moderate fat covering, patagium thickened with fat deposit; (**e**) Score 5, pronounced subcutaneous fat at neck, chest, and abdominal region, ribs not palpable with substantial fat covering, patagium thickened with substantial fat deposit.

The study period was from December 2019 to November 2021. Collection sites were three exotic pet veterinary practices in Taiwan. The protocol of this study was approved by the Institutional Animal Care and Use Committee of National Pingtung University of Science and Technology (NPUST-108-063; 16 December 2019). Sugar gliders were recruited via posters and social media posts. Recruited sugar gliders were normal in spirit, appetite, urination, and defecation, and showed no signs of estrus for the past two weeks. Signs of estrus in female sugar gliders may include increased barking, changes in temperament, increased interest from male sugar gliders, etc. Sugar gliders were not on any medication and did not have major medical conditions in the past. Clinical examination by a veterinarian was performed, and sugar gliders were determined clinically healthy. Clinical examination included attitude, gait, hydration status, external examination, auscultation, and palpation. In some cases, sugar gliders were not accustomed to handling, and detailed auscultation and palpation were performed during anesthesia. Body condition of sugar gliders were scored based on a chart derived from clinical experience and published papers of other species [16,17] (Figure 1). The score was 1 (thin) to 5 (obese) and assessed primarily by amount of fat palpated at the ribs and excess fat at the neck, chest, and patagium; individuals with scores of 1 or 5 were excluded from the study.

### 2.2. Anesthesia, Physiological Data and Morphometric Measurements

Sugar gliders were fasted for 4 h before examination. Oxygen and heat supplementation were initiated before anesthesia. Isoflurane (Halocarbon Isoflurane USP Liquid for Inhalation, Peachtree Corners, GA, USA) was used for induction (3% to 5%) and maintenance (1% to 3%) of anesthesia. Oxygen was maintained at a flow rate of 0.7 to 1 L/min. Respiratory rate, heart rate, pulse oximetry (Nonin PalmSAT^®^ 2500A, Plymouth, MN, USA), and reflexes were monitored during anesthesia. For nervous sugar gliders, parts of the clinical examinations were completed during anesthesia. After sample collection and all physiological and morphometrical values were recorded, isoflurane was discontinued, and oxygen and heat supplementation was provided until sugar gliders fully recovered. The anesthesia time of this study averaged around 20 min.

Physiological data, including anesthetized heart rate, anesthetized respiratory rate, auricular temperature, and cloacal temperature, were recorded. Heart rate and respiratory rate were obtained by auscultation and visual observation, respectively. Auricular temperature was obtained by infrared thermometer (Braun Thermoscan IRT 6520, Kronberg, Germany); cloacal temperature was obtained with digital oral thermometers (Rex Digital Thermometer MT-B182, Changhua, Taiwan; Valeo Digital Clinical Thermometer VT-801, Taipei, Taiwan). Morphometrical data were recorded by Vernier calipers; the data include body length (tip of snout to cloaca), tail length (cloaca to tip of tail), head length (tip of snout to occiput), head width (widest width across the zygomatic arches), right calf length (knee to heel), right foot length (heel to claw base), and lower incisor length (tip of lower incisor to gingival margin) [18]. If auricular and cloacal temperature cannot be accurately measured in some sugar gliders because of small body size, these data would not be collected. If the heart rate and respiratory rate of the anesthetized sugar glider were decreasing during data collection, the veterinarian would decide to discontinue the anesthesia, in which case some physiological and morphometrical values would not be measured.

### 2.3. Blood Sample Collection and Profile

**Figure 2:**
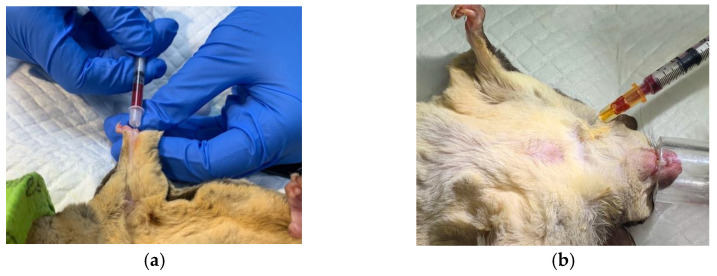
Two of the blood collection sites used in this study. (**a**) Median artery, using 0.3 mL insulin syringe; (**b**) cranial vena cava, using 1 mL syringe.

Blood samples were collected from the median artery, coccygeal artery, and cranial vena cava (Figure 2). Blood samples were collected with 0.3 or 0.5 mL insulin syringes (BD Ultra-Fine Insulin Syringes, Franklin Lakes, NJ, USA), as well as 1 mL syringes (BD 1 mL Syringe Slip Tip with BD 26G PrecisionGlide^TM^ Needle, Franklin Lakes, NJ, USA). Whole blood samples of 0.1 to 0.15 mL were placed in potassium EDTA tubes (potassium EDTA 0.5 mL Anticoagulant Tube, Vet Supply Global Co., Ltd., New Taipei, Taiwan) and 0.15 to 0.2 mL in heparin tubes (EV 0.5 mL Lithium Heparin Tube, Hebei Xinle Sci&Tech Co., Ltd., Hebei, China). The potassium EDTA tubes each had around 1.4 mg of potassium EDTA, while the heparin tubes had 10 IU of heparin. Hematological and plasma biochemical tests were performed within 2 h after sample collection.

Hematological parameters were examined with IDEXX ProCyte Dx^TM^ Hematology Analyzer (IDEXX Laboratories Inc., Westbrook, ME, USA), including: white blood cells (WBC); absolute and relative numbers of neutrophil (Neu; %Neu), lymphocyte (Lym; %Lym), monocyte (Mono; %Mono), eosinophil (Eos; %Eos), and basophil (Baso; %Baso); red blood cell count (RBC); hematocrit (HCT); hemoglobin (HGB); mean corpuscular volume (MCV); mean corpuscular hemoglobin (MCH); mean corpuscular hemoglobin concentration (MCHC); absolute and relative numbers of reticulocyte (Reti; %Reti); red blood cell distribution width (RDW); platelet count (Platelet); mean platelet volume (MPV); platelet distribution width (PDW); and plateletcrit (PCT). Thin blood slides were stained with a modified Romanowsky stain, Liu’s stain, formulated in the clinical pathology laboratory of the Veterinary Medical Teaching Hospital of National Pingtung University of Science and Technology. Blood slides were examined at x1000 magnification oil immersion with Olympus CX31 microscope (Olympus Corporation, Tokyo, Japan). Blood cell morphology was recorded with ISCapture software (Tucsen Photonics, Fujian, China). Diameter range of erythrocyte was obtained by measuring 50 cells from blood slides of 15 randomly selected sugar gliders from this study. Diameter range of each leukocyte type, except basophil, was measured the same way.

Plasma Biochemistry was examined with VetScan VS2^®^ Analyzer (Zoetis Inc., Parsippany, NJ, USA) using VetScan VS2^®^ Preventive Care Profile Plus rotors. The analyzed parameter included blood urea nitrogen (BUN), creatinine (CRE), alanine transaminase (ALT), alkaline phosphatase (ALP), aspartate transaminase (AST), total bilirubin (TBIL), blood glucose (GLU), calcium (CA), total protein (TP), albumin (ALB), globulin (GLOB), sodium (NA), potassium (K), and chloride (CL).

### 2.4. Statistical Analysis

The mean, standard deviation, and 95% confidence interval were calculated for physiological and morphometrical data. The reference intervals of each hematological and plasma biochemical parameter were calculated using Reference Value Advisor (RVA) software, following the American Society for Veterinary Clinical Pathology (ASVCP) guidelines [19,20]. There were two individuals under one year old, and these data were not included in physiological and morphometrical data analysis and reference interval calculation. Data distribution of each hematological and plasma biochemical index were first tested with the Anderson–Darling test. Box–Cox or natural log transformation were used for data transformation. Parametric method was used to determine reference intervals for data that were normally distributed originally or after transformation. Robust method was used for data that did not have, and could not be, transformed into normal distribution. Following the ASVCP guidelines, as the study population were deemed clinically healthy by veterinarians, outlier removal was not part of the calculation of the reference values [20].

Univariate analysis and multiple regression modeling of blood parameters from all recruited sugar gliders were conducted with RStudio (RStudio 2021.09.1 + 372 “Ghost Orchid,” RStudio, Massachusetts, USA). Sugar glider were grouped by age, gender, neuter status, location, diet, caging arrangement, other pets in household, and season. Ages from 7 to 59 months were grouped as “under-5-years-old” (n = 27), while age group “5-years-and-older” were 60 to 84 months (n = 14); one sugar glider was of unknown age and was excluded from univariate analysis of age. Females (n = 15) and males (n = 27) were both included in this study. Female sugar gliders were not commonly neutered in Taiwan; therefore, no neutered females were recruited. In univariate analysis of neuter status, only “neutered” males (n = 16) and “intact” males (n = 11) were analyzed. Sugar gliders kept in locations north of the 23.5 degrees north latitude were grouped as “north” (n = 14), and otherwise as “south” (n = 28). Diets without formulated foods or diets overly emphasizing on certain foods, including meats, fruits, and vegetables, were grouped as “unbalanced” (n = 10), otherwise grouped as “balanced” (n = 32). For caging arrangement, the “solitary” group individuals were housed individually (n = 14), while “with-conspecific-cagemate” sugar gliders were housed with one or more other sugar gliders (n = 28). Other pets in household was grouped “predatory” when sugar gliders were kept in contact or in vicinity of other pets that were larger and would be considered as their predators, such as cats or dogs (n = 9), otherwise “non-predatory” (n = 33). Seasons were partitioned as follows: September to November as “fall” (n = 10), December to February as “winter” (n = 5), March to May as “spring” (n = 20), and June to August as “summer” (n = 7).

To perform univariate analysis of age, gender, neuter status, location, diet, caging arrangement, and other pets in household on parameters, Mann–Whitney *U* tests were utilized for non-Gaussian parameters. For these factors, unpaired *t* tests were used for Gaussian-distributed parameters; folded F tests were used to test the homogeneity of variances, and when the variances were not equal, Welch’s *t* test were applied. To evaluate significance of season on non-Gaussian distributed parameters, Kruskal–Wallis tests, with Dunn’s multiple comparison test, were applied. One-way ANOVA tests were used to analyze seasonal effects on Gaussian-distributed data, with Tukey’s HSD test for multiple comparison; Levene’s test was used to test the homogeneity of variances, and if variances were not equal, Welch’s ANOVA was used. A *p* value less than 0.05 was considered significant. Multiple regression models were built with the parameters as continuous dependent variables, and the groups, as described above, serving as independent variables. The stepwise procedure and Akaike information criterion (AIC) were utilized for variable selection. Reference variate of age was “under-5-years-old,” gender was “female,” location was “north,” neuter status was “intact,” diet was “balanced,” caging arrangement was “with-conspecific-cagemate,” other pets in household was “non-predatory,” and season was “fall.” Parameter estimate and *p* values were calculated for the intercept and each included variable.

## 3. Results

### 3.1. Physiological and Morphometrical Data 

A total of 42 pet sugar gliders were included in this study, including 15 females and 27 males; of the male sugar gliders, there were 16 neutered individuals. The test subjects were between 7 months and 7 years of age. The sugar gliders were pets presented for health checks in veterinary clinics in Taiwanese cities New Taipei, Taichung, and Kaohsiung. Physiological and morphometrical data were recorded as mean, standard deviation, and 95% confidence interval in Table 1. The two sugar gliders under 1 year of age were not part of the calculation of these data.

### 3.2. Blood Cell Morphology

**Figure 3:**
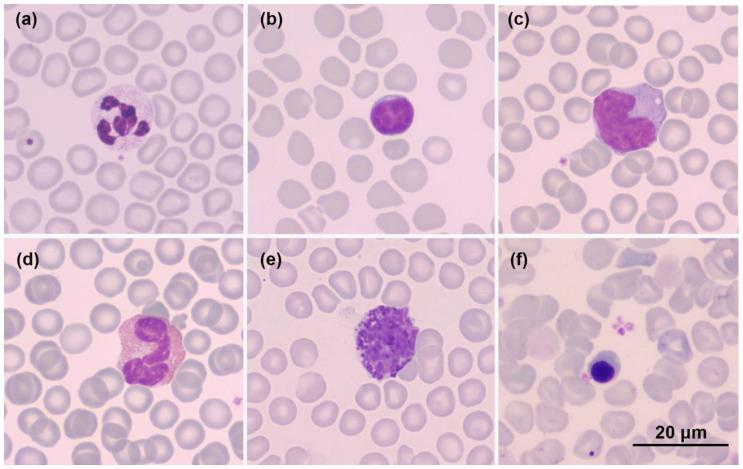
Blood cell morphology (Liu’s stain, x1000 magnification). (**a**) neutrophil; (**b**) lymphocyte; (**c**) monocyte; (**d**) eosinophil; (**e**) basophil; (**f**) nRBC (nucleated red blood cell).

Peripheral blood cell morphology was presented in Figure 3. Sugar glider red blood cells were anucleate cells with diameter ranging from 5.5 to 7.5 µm. Mild anisocytosis and polychromasia could be observed. Occasional Howell-Jolly bodies and nucleated RBCs were also present. Sugar glider neutrophil had segmented nucleus and pale cytoplasm, with diameter ranging from 11 to 14.5 µm; about 50 percent of neutrophil nuclei were observed to have 4 lobes, and 30 percent had 5 or more. Lymphocytes, predominantly small or medium lymphocytes, were the most abundant leukocytes on the blood slides of sugar gliders. The lymphocytes were 7.5 to 12.5 µm in diameter, had round, oval, or bean-shaped nucleus and a rim or band of pale to basophilic cytoplasm. Monocytes were 11 to 19.5 µm in diameter, had an irregular, sometimes horse-shoe shaped, nucleus and a substantial cytoplasm of blue-grey color with occasional vacuoles. Eosinophils ranged from 11.5 to 16.5 µm in diameter; the nuclei were 2 to 3 lobes, and the cytoplasm was densely populated with small, round eosinophilic granules. Basophils were sparsely observed in the blood slide examinations. Of the basophils measured, 10 to 17 µm diameter were recorded. Basophils had a segmented nucleus and deeply basophilic granules that were scattered loosely in the cytoplasm. Platelets were irregularly shaped and mostly stained lighter on the edge and darker in the middle.

### 3.3. Hematological and Plasma Biochemical Reference Values

The reference intervals for each parameter were summarized in Table 2 and Table 3. There were missing values in the hematological data set, due to analyzing errors that might have been caused by insufficient sample volume or sample influenced by prolonged blood drawing. There were missing values in the plasma biochemical data set, due to insufficient sample volume or moderate hemolysis. The hematological and plasma biochemical values of two sugar gliders under 1 year old were presented separately (Table A1 and Table A2) and were not part of the calculation of reference intervals. The reference range were presented as the lower limit and upper limit, with 95% confidence interval of both limits, as ASVCP guidelines suggested for reference intervals of smaller sample sizes [20].

### 3.4. Univariate Analysis

All significant univariate results were presented in Table 4. Relative lymphocyte count and ALB levels were significantly higher in the age group “5-years-and-older”, compared to “under-5-years-old,” while potassium and relative neutrophil count levels were higher in the older age group than the younger. Significant gender differences were found in RBC and CL. Male sugar gliders were found to have higher levels of RBC and CL, compared to female sugar gliders. None of the female sugar gliders in this study were neutered. Therefore, the effect of neuter status was only investigated among male sugar gliders in this study. Relative lymphocyte count, RBC, HCT, HGB, and RDW levels were found to be significantly higher for intact males, compared to neutered males, while the opposite was true for the MCH and GLOB levels. Significant location differences were found in levels of absolute and relative monocyte count, MCV, PCT, GLOB, and K. These parameters were all significantly higher in sugar gliders living in the south than the north. Levels of GLU displayed significant seasonal differences. Samples collected in the summer had significantly higher GLU values than in spring. Diet effect was observed, in that MCH levels were higher in the “balanced” group than in the “unbalanced” group. For the caging arrangement, whether sugar gliders were kept solitarily or not was not a significant factor in the univariate analyses. For the factor “Other Pets in Household,” between “predatory” and “non-predatory,” HGB, MCH, Reti, %Reti, and BUN levels were higher in “non-predatory” groups, while eosinophil counts were higher in “predatory” group.

### 3.5. Multiple Regression Model

Multiple regression models were built for each parameter of the hematological and plasma biochemical profile of pet sugar gliders in Taiwan (Table 5 and Table 6). All parameters, except for Mono, Eos, Baso, %Reti, PCT, CRE, and TBIL, had correlations with age. Notably, absolute and relative neutrophil counts and BUN, AST, GLOB, and potassium levels were higher in elder sugar gliders, while relative lymphocyte counts, ALB, NA and CL levels were lower. Regarding gender, the RBC, HCT, HGB, and RDW levels were higher in males than females, while MCH, MCHC, and %Reti were lower. Similarly, intact individuals had higher RBC, HCT, and HGB and lower MCH and MCHC than neutered individuals. Intact gliders also had higher platelet count and PCT. The location group “south” had higher absolute and relative monocyte counts, platelet, MPV, PCT, GLOB, and K than the group “north”. PDW, ALB and AST levels were higher in the group “north”, compared to “south”. Of the four seasons, the eosinophil concentration was lowest in the summer, reticulocyte concentration and percentage were highest in winter, BUN levels were highest in spring, and GLU was highest in summer. Animals in the “unbalanced” diet group had higher eosinophil count, RBC, platelet, and CL levels, while having lower MCH and MCV levels. Sugar gliders in the “solitary” group had lower percentage and concentration of reticulocytes, compared to animals in the “with-conspecific-cagemate” group. The “predatory” group of other pets in household factor had lower HGB and MCH levels, reticulocyte concentration and percentage, and BUN and ST levels, with higher platelet and PCT levels.

## 4. Discussion

This is the first study of sugar glider hematological and plasma biochemical reference interval in Asia. In the present study, the heart rate, respiratory rate, and cloacal temperature recorded during anesthesia were consistent with previous records of sugar gliders [4,21]. Auricular temperature was also recorded during anesthesia, and the mean auricular temperature was higher than the mean cloacal temperature recorded in this study (36.2 °C and 35.4 °C, respectively). In marsupials, small size can prohibit the measuring of auricular temperature, otherwise it had been suggested that auricular temperature was more accurate than cloacal temperature [22]. Morphometric measurements of this study were generally similar to data of wild sugar gliders, except that pet sugar gliders had higher mean body weight than wild sugar gliders [18,23]. In future sugar glider research, it would be valuable to collect physiological data of a larger sample size to expand on this preliminary data.

The morphological characteristics of peripheral blood cells in the present study, such as the staining and pattern of nuclei, granules, and cytoplasm, were consistent with previous records of sugar gliders and similar to other marsupials, such as squirrel gliders, koalas, and quokkas [24,25,26]. The nuclei of marsupial neutrophils typically have 3 to 6 lobes, and that of canine and feline neutrophil have 3 to 5 lobes [27,28,29]. Sugar gliders have a larger portion of neutrophils, with 5 or more nuclear lobes, which would be considered hypersegmentation in canine and feline hematology [30,31,32].

In the present study, the mean ALP value of adult sugar gliders was higher than previously reported in pet sugar gliders [6]. The increase in ALP values had been related to osteoblastic activity during growth in marsupials, and young individuals were found to have higher ALP values, same as the findings in this study [33,34,35]. The two sugar gliders below 1 year old had higher ALP levels than the calculated adult reference range. Other sources of ALP include the liver, kidneys, and intestines in mammals, such as dogs, cats, and rabbits; however, isoform profiles have not been studied in marsupials [11,36,37,38]. The mean CRE values in the present study were lower than formerly published values of pet sugar gliders [6,10]. It has been reported that lower CRE levels in healthy animals have been associated to lower muscle mass in quolls (*Dasyurus* spp.) and rodents, as well as hydration in dogs, cats, and rabbits [34,39,40,41,42]. Lower mean CRE value in this study could be related to the differences of diet consumption and smaller animal living space in Taiwan, compared to the United States, where the previous studies took place [5,43].

Based on the multiple regression model, levels of RBC, HCT, and HGB were higher in males and in intact sugar gliders. Higher RBC counts in males was also found in studies of tammar wallabies (*Macropus eugenii*), western ringtail possums (*Pseudocheirus occidentalis*), greater gliders (*Petauroides volans*), and allied rock-wallabies (*Petrogale assimilis*) [44,45,46,47]. Higher RBC, HGB, and HCT levels were also recorded in male common and mountain brushtail possums (*Trichosurus vulpecula* and *cunninghami*) [48,49,50]. RBC count was known to be stimulated by male sex hormones in mammals and could increase in rats and mice after testosterone injection, while decreasing in rats and hamsters after castration [44,51,52,53].

In the present study, sugar gliders’ ages ranged from 7 months to 7 years. Considering that the lifespan of wild sugar gliders is 4 to 6 years, sugar gliders 5 years or older were deemed the elder group [3,4]. In a study of captive Tasmanian devils (*Sarcophilus harrisii*), when the animals were older, living beyond their wild lifespan, changes in blood values, such as total protein, were observed [33]. In this study, relative lymphocyte count were lower in the elder group of sugar gliders, while relative neutrophil counts were higher, similar to the findings of the studies in Gilbert’s potoroos (*Potorous gilbertii*), Tasmanian devils, allied rock-wallabies, koalas (*Phascolarctos cinereus*), and eastern grey kangaroos (*Macropus giganteus*) [25,33,35,47,54,55]. There was also a similar phenomenon in the hematology of laboratory animals, such as rabbits and rodents [56,57,58]. Higher levels of lymphocyte in young animals were related to immune system development and first exposure to antigens in Gilbert’s potoroos, Tasmanian devils, and dogs, and the age effect of lymphocyte population was linked with sex hormones, glucocorticoids, and thymus involution in humans [35,54,59,60]. Increase in neutrophil levels had been related to cortisol increase, due to the stress of malnutrition or subclinical disease, in marsupials, as well as in eutherians [11,35,56].

Higher levels of AST in older animals were found in the present study, and similarly in older adult Tasmanian devils, compared to younger adults [33]. AST levels were associated with tissue damage of the liver, heart, and skeletal muscle [38,61]. ALB levels were lower in the elder group in this study, and similar trends had been found in Tasmanian devils and dogs, with an association with muscle loss, proposed in humans [36,54,62,63]. Subclinical degenerative change in the liver, heart, and muscle could lead to the age effect in AST and ALB.

Levels of GLOB and K were higher in sugar gliders living in the south of Taiwan than in the north. Previous marsupial studies had attributed locational differences to genetics, temperature, water availability, and diet quality [13,14,45]. Studies had linked higher values of GLOB and K to increases in environmental temperature in sheep and rabbits or stress in rodents [39,64,65]. The average temperature of Taipei in northern Taiwan was around 1.6 degrees Celsius lower than Kaohsiung in southern Taiwan during the study period (Central Weather Bureau, 2021). Pet sugar gliders are usually kept in indoor cages, with occasional out-of-cage activity indoors, and fed once or twice a day. As indoor pets, temperature effect would be less significant for pet sugar gliders in Taiwan. The effect of location in the present study could contain multiple environmental and husbandry factors, and the specific cause of higher GLOB and K could not be singled out.

Sugar gliders had higher GLU values in summer and higher BUN values in spring. Such seasonal GLU variations were also found in quolls and wild brushtail possums and were related to breeding, water availability, and changes in climate and environment [34,66]. Changes in glucose levels had also been linked to stressful events and excitement-induced catecholamine release [36,45,54]. Urea was also found to be higher in spring in wild Tasmanian devils and was considered to be influenced by the physiological demands of breeding [67]. Changes in BUN were often linked to various factors including hydration, protein intake, and exercise in healthy domestic mammals [36,42]. Association with breeding activity was not considered in this study, since sugar gliders during estrus were excluded. Seasonal changes in the environment, such as temperature, humidity, and circadian rhythm, and being active for longer periods of time in spring and summer might be related to the seasonal differences in GLU and BUN levels in the present study; however, due to small sample size in the season groups, this preliminary result needs to be studied on a larger scale.

In the experimental research of captive sugar gliders, the diet effects on blood parameters of these animals were studied [15]. Nine sugar gliders were divided into three groups and, in addition to assorted fruits and vegetables, fed one of three commonly available diets: Insectivore Fare from Reliable Protein Products, soaked kibble from Eight in One Pet Products, and Bourbon’s Modified Leadbeater’s diet [15]. Mineral imbalances in the analyzed diets were found, and higher BUN and lower CA levels were found between the groups; however, the significance of the diet’s influence on blood values could not be established, due to the small sample size of the study [15]. In the present study, diets with over 30% fruits, vegetables, or animal-based protein, as well as diets without formulated feeds, were considered unbalanced. Excess in fruits, vegetables, and animal-based protein in the diet of sugar gliders had been linked with obesity, periodontal disease, and metabolic bone disease, and formulated feeds, such as Mazuri insectivore diet and Leadbeater’s recipe, were often recommended [68,69,70,71]. In the present study, higher eosinophil and platelet counts and lower MCV levels were found in sugar gliders with unbalanced diets. Eosinophil level changes were generally linked to parasitic infection and hypersensitivity in domestic mammals [36,72]. Increased platelet count could be related to inflammation, hemorrhage, or iron-deficiency anemia in marsupials [24]. Increases in platelet levels were linked with iron deficiency in humans and dietary imbalance in cats [73,74]. Decrease in MCV was mostly linked to the microcytic anemia caused by iron deficiency in dogs and cats [36,75]. The sugar gliders in this study did not have signs of infection, hypersensitivity, or anemia. Hematological values could have been influenced by the diets of pet sugar gliders, yet further research is needed for a more definite conclusion.

Being social animals in the wild, keeping sugar gliders in pairs or groups was thought to be necessary, for fear of stress from lacking social interactions [9,43]. Solitary sugar gliders were described to be prone to aggression and self-mutilation; however, solitary sugar gliders in the present study were healthy, and these behaviors were not observed [9,43]. There were no major hematological or plasma biochemical differences between sugar gliders kept in solitary or with conspecifics in this study.

In the wild, sugar gliders were considered prey animals. Some pet sugar gliders in this study were kept in the same household as pets considered predatory animals, such as dogs and cats. Behavioral changes due to the scent of predators were documented in captive marsupial literature, but physiological changes were not yet studied [76,77]. BUN and HGB levels were lower for sugar gliders kept with predatory pets. BUN was mostly related to protein intake in healthy animals [45,78]. Differences in erythrocyte parameters, such as HGB, were also linked to nutrition and body condition [24,33]. Stressful environments, due to living around predatory animals, may have affected feeding habits and hindered the consumption and absorption of nutrients. However, more evidence and information are needed in this aspect for a definite conclusion.

## 5. Conclusions

The hematological and plasma biochemical reference interval, as well as physiological and morphometrical data of pet sugar gliders in Taiwan, were presented in this study. The possible effects of age, gender, neuter status, location, season, diet, caging arrangement, and other pets in the household were also provided, with the effect of gender on erythrocyte values and effect of age on leukocytes particularly notable. This was the first study on the effect of physiological and environmental factors on the blood profile of pet sugar gliders, yet many aspects of this topic are still unknown, and further investigation is needed. The reference interval and other data compiled in this study can be useful for veterinarians and beneficial to sugar glider medicine.

## Figures and Tables

**Table 1:** Physiological data and morphometric measurements of sugar gliders, including mean and standard deviation.

	Units	Mean	Standard Deviation	n
Physiological data				
Respiratory rate (conscious)	breaths/min	136.43	71.42	14
Respiratory rate (anesthetized)	breaths/min	33.79	18.63	39
Cloacal temperature ^a^	°C	35.40	0.91	34
Auricular temperature ^a^	°C	36.20	0.78	22
Heart rate (conscious)	beats/min	306.05	42.38	19
Heart rate (anesthetized)	beats/min	244.50	46.76	40
Morphometric measurements				
Body weight	g	144.07	34.68	40
Body length	mm	145.70	6.72	40
Tail length	mm	160.36	18.25	40
Head width	mm	26.24	2.05	40
Head length	mm	38.25	2.39	40
Right calf length	mm	41.69	2.14	40
Right foot length	mm	23.89	1.97	39
Lower incisor	mm	6.51	0.54	39

^a^ Temperature data recorded while anesthetized.

**Table 2:** Hematological reference intervals of pet sugar gliders in Taiwan.

Parameters	Unit	n	Mean	SD	Lower Limit (90% CI)	Upper Limit (90% CI)	Distribution	Method
RBC	M/µL	39	8.03	0.90	6.18 (5.80–6.58)	9.88 (9.46–10.29)	G	P
HCT	%	39	46.30	5.03	35.99 (33.88–38.22)	56.62 (54.27–58.86)	G	P
HGB	g/dL	39	16.56	1.65	13.18 (12.49–13.91)	19.93 (19.16–20.67)	G	P
MCV	fL	39	57.77	3.44	50.72 (49.29–52.25)	64.82 (63.22–66.35)	G	P
MCH	pg	39	20.67	1.10	18.41 (17.95–18.90)	22.92 (22.41–23.42)	G	P
MCHC	g/dL	39	35.81	1.43	32.87 (32.27–33.51)	38.74 (38.08–39.38)	G	P
Reti	K/µL	39	242.49	60.42	118.61 (93.34–145.35)	366.37 (338.22–393.33)	G	P
%Reti	%	39	3.00	0.68	1.82 (1.64–2.05)	4.62 (4.17–5.05)	BG	P
RDW	%	39	22.95	2.46	17.90 (16.87–18.99)	27.99 (26.84–29.09)	G	P
WBC	K/µL	33	7.80	2.95	1.71 (0.37–3.14)	13.89 (12.38–15.33)	G	P
Neu	K/µL	33	1.44	0.94	0.35 (0.27–0.49)	4.25 (3.03–5.67)	BG	P
%Neu	%	33	17.92	6.92	7.72 (6.51–9.25)	36.28 (29.96–43.58)	LG	P
Lym	K/µL	33	5.79	2.22	1.20 (0.18–2.04)	10.39 (9.25–11.48)	G	P
%Lym	%	33	74.54	7.77	58.84 (54.93–62.25)	90.60 (86.63–96.41)	G	P
Mono	K/µL	33	0.36	0.19	0.06 (0.03–0.11)	0.84 (0.68–0.98)	BG	P
%Mono	%	33	4.59	2.03	0.40 (0–1.38)	8.78 (7.75–9.78)	G	P
Eos	K/µL	33	0.14	0.11	0.03 (0.02–0.04)	0.52 (0.33–0.76)	BG	P
%Eos	%	33	1.95	1.38	0.26 (0.18–0.44)	5.88 (4.34–7.45)	BG	P
Baso	K/µL	33	0.07	0.05	0 (0–0)	0.18 (0.15–0.20)	G	P
%Baso	%	33	1.01	0.75	0 (0–0)	2.45 (1.87–3.07)	NG	Ro
Platelet	K/µL	39	385.70	144.10	90.20 (29.90–154.00)	681.10 (614.00–745.40)	G	P
MPV	fL	39	8.57	1.14	6.23 (5.75–6.74)	10.92 (10.39–11.43)	G	P
PDW	fL	39	8.67	1.36	5.88 (5.32–6.48)	11.45 (10.82–12.06)	G	P
PCT	%	39	0.33	0.14	0.14 (0.12–0.17)	0.70 (0.56–0.87)	BG	P

SD: standard deviation; CI: confidence interval; G: Gaussian; BG: Box–Cox transformed Gaussian; LG: natural log transformed Gaussian; NG: non-Gaussian; P: parametric; Ro: robust.

**Table 3:** Plasma biochemical reference intervals of pet sugar gliders in Taiwan.

Parameters	Unit	n	Mean	SD	Lower Limit (90% CI)	Upper Limit (90% CI)	Distribution	Method
BUN	mg/dL	39	22.50	8.00	9.60 (8.00–11.70)	42.40 (36.70–48.20)	BG	P
CRE	mg/dL	39	0.27	0.14	0 (0–0.04)	0.58 (0.52–0.63)	NG	Ro
ALT	U/L	39	79.40	39.50	24.90 (20.40–31.70)	189.40 (153.60–227.50)	BG	P
ALP	U/L	39	174.20	82.50	68.90 (58.06–82.59)	369.13 (305.05–443.11)	LG	P
AST	U/L	38	48.00	19.30	5.10 (0–15.00)	85.60 (72.30–98.70)	NG	Ro
TBIL	mg/dL	39	0.44	0.24	0 (0–0)	0.87 (0.76–0.97)	NG	Ro
GLU	mg/dL	39	158.00	29.90	86.10 (73.60–103.00)	213.00 (196.00–234.30)	NG	Ro
CA	mg/dL	39	8.98	0.69	7.57 (7.28–7.88)	10.40 (10.08–10.71)	G	P
TP	g/dL	39	6.09	0.55	4.95 (4.72–5.20)	7.23 (6.97–7.47)	G	P
ALB	g/dL	39	4.35	0.50	3.34 (3.13–3.56)	5.37 (5.14–5.59)	G	P
GLOB	g/dL	39	1.74	0.51	0.69 (0.48–0.92)	2.78 (2.54–3.01)	G	P
NA	mmol/L	39	138.80	3.20	132.30 (130.90–133.70)	145.40 (143.90–146.80)	G	P
K	mmol/L	37	3.86	0.62	2.59 (2.32–2.87)	5.13 (4.83–5.41)	G	P
CL	mmol/L	38	106.90	3.50	99.80 (98.30–101.30)	114.10 (112.50–115.70)	G	P

SD: standard deviation; CI: confidence interval; G: Gaussian; BG: Box–Cox transformed Gaussian; LG: natural log transformed Gaussian; NG: non-Gaussian; P: parametric; Ro: robust.

**Table 4:** Significant statistical test results of univariate analysis on hematological and plasma biochemical parameters.

Factors	Parameter	Test	Result	*p* Value
Age	%Lym	*t* test	[<5 years] > [≥5 years]	0.01292
	K	*t* test	[≥5 years] > [<5 years]	0.00219
	%Neu	Mann–Whitney *U* test	[≥5 years] > [<5 years]	0.02806
	ALB	Mann–Whitney *U* test	[<5 years] > [≥5 years]	0.04936
Gender	RBC	*t* test	Male > Female	0.04133
	CL	*t* test	Male > Female	0.03229
Neuter Status Of Males	RBC	Welch’s *t* test	Intact > Neutered	0.00058
	HCT	*t* test	Intact > Neutered	0.03444
	HGB	Welch’s *t* test	Intact > Neutered	0.02086
	MCH	*t* test	Neutered > Intact	0.00788
	%Lym	*t* test	Intact > Neutered	0.03538
	GLOB	*t* test	Neutered > Intact	0.00947
	RDW	Mann–Whitney *U* test	Intact > Neutered	0.03180
Location	MCV	*t* test	South > North	0.03581
	%Mono	Welch’s *t* test	South > North	0.00329
	GLOB	*t* test	South > North	0.00515
	K	*t* test	South > North	0.03729
	Mono	Mann–Whitney *U* test	South > North	0.03639
	PCT	Mann–Whitney *U* test	South > North	0.01455
Season	GLU	Kruskal–Wallis test Post hoc Dunn’s test	Summer > Spring	0.00375
Diet	MCH	*t* test	Balanced > Unbalanced	0.00386
Other Pets in Household	HGB	*t* test	Non-predatory > Predatory	0.02660
	MCH	*t* test	Non-predatory > Predatory	0.03165
	Reti	*t* test	Non-predatory > Predatory	0.02251
	%Reti	*t* test	Non-predatory > Predatory	0.04382
	Eos	Mann–Whitney *U* test	Predatory > Non-predatory	0.04986
	BUN	Mann–Whitney *U* test	Non-predatory > Predatory	0.00448

**Table 5:** Results of multiple regression models of hematological parameters.

	Intercept	Age		Gender		Neuter Status		Location		Diet		Caging Arrangement		Other Pets in Household		Season (Spring)		Season (Summer)		Season (Winter)	
Parameters	PE	PE	*p* Value	PE	*p* Value	PE	*p* Value	PE	*p* Value	PE	*p* Value	PE	*p* Value	PE	*p* Value	PE	*p* Value	PE	*p* Value	PE	*p* Value
RBC	7.327	−0.134	0.586	1.291	**<0.001**	−1.250	**<0.001**	0.398	0.099	0.776	**0.006**	-	-	-	-	-	-	-	-	-	-
HCT	42.417	−0.235	0.883	4.945	**0.016**	−5.958	**0.003**	2.938	0.062	2.960	0.097	2.766	0.092	-	-	-	-	-	-	-	-
HGB	16.301	0.259	0.626	1.844	**0.005**	−1.589	**0.013**	-	-	-	-	-	-	−1.401	**0.018**	-	-	-	-	-	-
MCV	57.049	1.393	0.212	-	-	-	-	1.620	0.148	−2.502	0.050	-	-	-	-	-	-	-	-	-	-
MCH	21.200	0.449	0.132	−0.931	**0.009**	1.000	**0.005**	-	-	−1.161	**0.001**	-	-	−0.908	**0.007**	-	-	-	-	-	-
MCHC	35.608	0.107	0.830	−0.939	0.122	1.490	**0.013**	−0.902	**0.049**	-	-	-	-	-	-	1.462	**0.010**	−0.201	0.770	−0.486	0.548
Reti	249.681	−18.155	0.382	-	-	-	-	-	-	-	-	−37.572	0.089	−67.193	**0.008**	16.617	0.489	2.485	0.936	108.036	**0.003**
%Reti	3.225	-	-	−0.389	0.081	-	-	-	-	-	-	−0.500	**0.045**	−0.757	**0.006**	0.270	0.318	0.099	0.762	1.318	**0.001**
%RDW	22.546	−0.909	0.378	3.425	**0.008**	−2.854	**0.024**	-	-	-	-	-	-	-	-	-	-	-	-	-	-
WBC	7.880	0.750	0.538	-	-	-	-	-	-	-	-	-	-	-	-	-	-	-	-	-	-
Neu	1.397	0.767	**0.035**	-	-	-	-	-	-	-	-	−0.536	0.142	-	-	-	-	-	-	-	-
%Neu	16.491	7.218	**0.004**	-	-	-	-	-	-	-	-	−3.580	0.146	-	-	-	-	-	-	-	-
Lym	6.045	0.012	0.989	-	-	-	-	-	-	-	-	-	-	-	-	-	-	-	-	-	-
%Lym	77.871	−6.401	**0.020**	-	-	-	-	-	-	−3.893	0.170	-	-	-	-	-	-	-	-	-	-
Mono	0.274	-	-	-	-	-	-	0.141	**0.044**	-	-	-	-	-	-	-	-	-	-	-	-
%Mono	2.814	0.519	0.455	-	-	1.086	0.109	1.800	**0.012**	-	-	-	-	-	-	-	-	-	-	-	-
Eos	0.190	-	-	-	-	-	-	-	-	0.104	**0.024**	-	-	-	-	−0.061	0.175	−0.165	**0.007**	−0.098	0.120
%Eos	1.698	0.148	0.772	-	-	-	-	-	-	0.847	0.125	-	-	-	-	-	-	-	-	-	-
Baso	0.073	-	-	-	-	-	-	-	-	-	-	-	-	-	-	-	-	-	-	-	-
%Baso	0.854	−0.148	0.595	-	-	0.426	0.119	-	-	-	-	-	-	-	-	-	-	-	-	-	-
Platelet	335.717	−26.523	0.569	-	-	−126.445	**0.006**	105.634	**0.028**	112.846	**0.030**	-	-	81.028	0.157	-	-	-	-	-	-
MPV	8.198	−0.334	0.382	-	-	-	-	0.859	**0.029**	-	-	-	-	-	-	-	-	-	-	-	-
PDW	9.434	−0.710	0.124	-	-	-	-	−0.815	0.079	-	-	-	-	-	-	-	-	-	-	-	-
PCT	0.247	-	-	-	-	−0.119	**0.018**	0.149	**0.007**	0.076	0.188	-	-	0.123	**0.040**	-	-	-	-	-	-

PE: parameter estimate. Reference variate of Age was under-5-years-old, Gender was female, Location was north, Neuter Status was intact, Diet was balanced, Caging Arrangement was with-conspecific-cagemate, Other Pets in Household was non-predatory, Season was fall.

**Table 6:** Results of multiple regression models of plasma biochemical parameters.

	Intercept	Age		Gender		Neuter Status		Location		Diet		Caging Arrangement		Other Pets in Household		Season (Spring)		Season (Summer)		Season (Winter)	
Parameters	PE	PE	*p* Value	PE	*p* Value	PE	*p* Value	PE	*p* Value	PE	*p* Value	PE	*p* Value	PE	*p* Value	PE	*p* Value	PE	*p* Value	PE	*p* Value
BUN	20.906	3.473	0.186	-	-	-	-	−3.686	0.147	-	-	-	-	−6.279	0.052	6.916	0.023	3.268	0.384	2.095	0.613
CRE	0.273	-	-	-	-	-	-	-	-	-	-	-	-	-	-	-	-	-	-	-	-
ALT	68.464	14.898	0.254	-	-	-	-	-	-	20.987	0.147	-	-	-	-	-	-	-	-	-	-
ALP	162.574	−23.672	0.544	62.672	0.114	-	-	-	-	-	-	-	-	-	-	-	-	-	-	-	-
AST	49.644	21.042	**0.001**	-	-	-	-	−11.622	0.066	-	-	-	-	−10.724	0.132	-	-	-	-	-	-
TBIL	0.463	-	-	-	-	-	-	-	-	-	-	-	-	−0.118	0.177	-	-	-	-	-	-
GLU	152.579	−1.198	0.907	-	-	-	-	-	-	-	-	-	-	-	-	−9.906	0.384	39.487	**0.011**	19.579	0.231
CA	9.058	0.207	0.370	-	-	-	-	-	-	−0.421	0.102	-	-	-	-	-	-	-	-	-	-
TP	6.385	−0.282	0.107	−0.268	0.126	-	-	-	-	-	-	-	-	-	-	-	-	-	-	-	-
ALB	4.713	−0.447	**0.003**	-	-	-	-	−0.291	**0.049**	-	-	-	-	-	-	-	-	-	-	-	-
GLOB	1.354	0.206	0.175	-	-	-	-	0.494	**0.002**	-	-	-	-	-	-	-	-	-	-	-	-
NA	139.654	−2.264	**0.033**	-	-	-	-	-	-	−1.742	0.138	-	-	-	-	0.690	0.544	−1.862	0.224	2.802	0.100
K	3.176	0.825	**<0.001**	-	-	-	-	0.424	**0.017**	-	-	-	-	-	-	0.287	0.156	0.329	0.204	−0.330	0.249
CL	104.773	−2.261	0.075	-	-	-	-	1.739	0.160	3.438	**0.020**	-	-	-	-	2.243	0.100	−2.183	0.263	3.529	0.081

PE: parameter estimate. Reference variate of Age was under-5-years-old, Gender was female, Location was north, Neuter Status was intact, Diet was balanced, Caging Arrangement was with-conspecific-cagemate, Other Pets in Household was non-predatory, Season was fall.

## Data Availability

Not applicable.

## Appendix A

**Table A1:** Hematology data of two sugar gliders under one year old. Missing data was caused by errors in the analyzing process, possibly due to insufficient sample volume.

Parameters	Unit	SG1	SG2
WBC	K/µL	16.00	-
Neu	K/µL	2.79	-
%Neu	%	17.4	-
Lym	K/µL	12.13	-
%Lym	%	75.8	-
Mono	K/µL	0.66	-
%Mono	%	4.1	-
Eos	K/µL	0.36	-
%Eos	%	2.3	-
Baso	M/µL	0.06	-
%Baso	%	0.4	-
RBC	g/dL	9.17	8.47
HCT	fL	51.7	54.0
HGB	pg	18.5	17.3
MCV	g/dL	56.4	63.8
MCH	K/µL	20.2	20.4
MCHC	%	35.8	32.0
Reti	%	237.5	118.6
%Reti	K/µL	2.6	1.4
%RDW	fL	29	34.8
Platelet	%	201	870
MPV	%	-	10.6
PDW	K/µL	-	-
PCT	%	-	0.92

SG1: an eight-month-old sugar glider, SG2: a seven-month-old sugar glider.

**Table A2:** Plasma biochemical data of two sugar gliders under one year old.

Parameters	Unit	SG1	SG2
BUN	mg/dL	18	17
CRE	mg/dL	0.2	0.3
ALT	U/L	72	40
ALP	U/L	501	617
AST	U/L	41	0
TBIL	mg/dL	0.4	0.4
GLU	mg/dL	156	76
CA	mg/dL	9.2	9.8
TP	g/dL	6.4	5.7
ALB	g/dL	4.4	4.3
GLOB	g/dL	2	1.5
NA	mmol/L	140	136
K	mmol/L	3.8	4.3
CL	mmol/L	118	108

SG1: an eight-month-old sugar glider, SG2: a seven-month-old sugar glider, CA: calcium, NA: sodium, K: potassium, CL: chloride.
